# Remembering Thomas C. Rindflesch: a journey from language to biomedical discovery

**DOI:** 10.1093/jamiaopen/ooag187

**Published:** 2026-09-23

**Authors:** Halil Kilicoglu, Marcelo Fiszman, Graciela Rosemblat, François-Michel Lang, Anna Ripple, Olivier Bodenreider

**Affiliations:** School of Information Sciences, University of Illinois Urbana-Champaign, Champaign, IL, United States; Semedy, Inc, Newton, MA, United States; Greenfort University, Orlando, FL, United States; Retired, Formerly U.S. National Library of Medicine, Bethesda, MD, United States; Retired, Formerly U.S. National Library of Medicine, Bethesda, MD, United States; Retired, Formerly U.S. National Library of Medicine, Bethesda, MD, United States; Retired, Formerly U.S. National Library of Medicine, Bethesda, MD, United States

**Keywords:** natural language processing, semantics, literature-based discovery, mentorship, obituary

Thomas (“Tom”) C. Rindflesch (1947-2026), PhD, FACMI, died on March 20, 2026, after a brief battle with cancer. A research scientist at the US National of Medicine (NLM) from 1991 to 2017 and long-time lead of the Semantic Knowledge Representation (SKR) project, Tom was one of the pioneers of biomedical natural language processing (NLP). His contributions, spanning concept recognition, relation extraction, knowledge representation, and literature-based discovery (LBD), were foundational to the field over more than two decades and left behind tools and resources that the community continues to use and build upon today.

A native of Minnesota, Tom earned his BA in Arabic before pursuing graduate study in linguistics at the University of Minnesota, where he completed a PhD under the supervision of Michael Kac. His dissertation, *Linguistic Aspects of Natural Language Processing*,^1^ argued that natural-language parsers should be grounded in the empirical structure of language as it is used, rather than formal language theories developed around mathematical properties of formal languages, the dominant paradigm at the time, which reflected his lifelong commitment to linguistic structure as a foundation for NLP for the rest of his career.

NLM hired him in 1991 to adapt PUNDIT,^2^ a natural language understanding system combining syntactic and semantic processing, to biomedical text to support automated indexing and biomedical language understanding. This was before the time full syntactic parsers were sufficiently accurate and, by his own account, adapting one to the biomedical domain proved intractable because of the combinatorial explosion of PUNDIT’s syntactic analyses. Tom pivoted to developing what he called the *minimal commitment parser*,^3^ a shallow parsing approach drawing on his dissertation work, that analyzed noun phrase structure and subordination relations rather than attempting full syntactic parsing. The key insight was that partial analysis, focused on the structures most relevant to semantic interpretation, was both more tractable and more useful for downstream tasks.

Minimal commitment parser became the core syntactic analysis module of MetaMap,^4^^,^^5^ NLM’s widely used biomedical concept recognition tool. Tom also developed the linguistically inspired scoring mechanisms, namely *centrality*, *variation*, *coverage*, and *cohesiveness*, used to rank candidate concepts, which have remained part of MetaMap’s design ever since.

Tom’s central and most sustained contribution (“his child”) was SemRep,^6^^,^^7^ a broad-coverage, linguistically based system for extracting semantic relations from biomedical text in the form of subject-predicate-object triples. Building on MetaMap’s concept recognition, the UMLS Semantic Network,^8^ and a handful of one-off relation extraction studies, such as EDGAR,^9^ SemRep unified a body of earlier work on biomedical relation extraction in the early 2000s.^6^ Its defining innovations included *indicator rules* (triggering rules that map lexical items to Semantic Network relations), along with argument sharing principles, empty head marking, and first-order logical inference, all implemented in Prolog, the programming language Tom mastered—and the only one he ever felt the need to. Subsequent development extended SemRep to handle nominalizations, hypernymy, anaphora, and specialized language of molecular biology. From a theory standpoint, SemRep drew on lexical semantics,^10^ ontological semantics,^11^ and Meaning-Text Theory,^12^ while grounding domain knowledge in the UMLS.^13^

Tom’s approach was deliberately incremental and linguistically principled, in deliberate contrast to the statistical and neural methods that came to dominate NLP since the 1990s. He believed that systematic attention to linguistic phenomena, combined with improvements in the UMLS, would ultimately yield systems capable of practical, broad-coverage biomedical text understanding, a goal that remains unfulfilled even today. Although large language models have shown strong results across a range of biomedical tasks, producing reliable, interpretable, and normalized semantic representations at scale remains a significant challenge, particularly for systems intended to support formal knowledge representation and inference. In this respect, his skepticism appears to have been well founded. Characteristically, he never published a single comprehensive SemRep paper, his linguistic perfectionism made such a synthesis impossible to finalize, though a more practical overview appeared with his blessing after his retirement.^7^

Bringing SemRep to PubMed scale transformed it from a tool for individual texts into an infrastructure for discovery, beginning with graph-based literature summarization^14^ to the Semantic MEDLINE tool^15^^,^^16^ and ultimately to SemMedDB.^17^ SemMedDB is a large-scale biomedical knowledge graph of semantic triples extracted from PubMed, a knowledge graph, it is worth noting, before Google popularized the term around 2012. In its final version, released by NLM in 2024, SemMedDB contained more than 130M triples and has supported a wide range of clinical and translational applications: drug repurposing, clinical guideline development, medical diagnosis, clinical decision support, and LBD. For example, it was incorporated as a core knowledge source in the NCATS Translator program^18^^,^^19^ and has been credited as a key resource in drug repurposing cases for rare diseases. Semantic MEDLINE extended SemMedDB with graph-based summarization and interactive visualization, allowing users to navigate biomedical literature not as a list of documents but as a network of concepts and relationships, a genuinely visionary approach to knowledge management and hypothesis generation that anticipated current interest in graph-based biomedical AI, discovery, and network medicine.

From the mid-2000s onward, Tom and his collaborators made substantial contributions to LBD, the field concerned with generating novel hypotheses by linking findings across disconnected bodies of literature. Beginning with collaborations with Dimitar Hristovski of the University of Ljubljana, Tom extended Don Swanson’s A-B-C paradigm into a sustained research program encompassing discovery patterns, analogical reasoning using vector semantics, discovery browsing, and link prediction/knowledge graph completion methods, all using semantic triples as the foundation. For example, his group used Semantic MEDLINE, combined with expert interpretation, to propose a possible link between age-related hypogonadism and diminished sleep quality,^20^ and later applied discovery browsing approaches to investigate possible biological explanations for the observed association between inflammatory bowel disease and epilepsy, including the role of IL-1β and glutamate.^21^ A scientometric analysis of LBD research identified Tom as one of two central hubs of the field.^22^ He liked to say that whatever biomedical expertise he had acquired had grown out of countless hours spent playing with Semantic MEDLINE.

Tom occupied an unusual position in biomedical informatics, a committed linguist in a field that had largely moved on from linguistics. Where others embraced statistical and probabilistic models, he remained convinced that meaning was fundamentally ontological rather than statistical. He was fond of quoting Sergei Nirenburg’s formulation that “frequency of occurrence is an epiphenomenon of meaning,” a phrase that neatly captured his research perspective and that he deployed with relish in arguments about semantics and NLP. He cared about ideas, rigorous work, and results that mattered in practice and was less interested in incremental performance gains on narrow datasets or publishing prolifically. That he spent his career as a government scientist, largely free from the funding pressures that shape most research agendas, gave him the rare freedom to pursue this vision on his own terms, a reminder of what curiosity-driven research can look like. While his views might have been in the minority in a field moving steadily toward benchmark-driven evaluation, his research contributions were still recognized with his election as an ACMI Fellow in 2005, an AMIA Distinguished Paper Award in 2007,^23^ and the NLM Board of Regents Award in 2017. He described himself as something of an “acquired taste” in the community and did not much mind being one.

Tom was, by the testimony of nearly everyone who worked with him, an exceptional mentor. He received the NIH Director’s Award in 2015 for a mentoring record that spanned high school students, undergraduates, graduate students, postdoctoral fellows, and visiting scholars, as well as the informal guidance he offered to more established researchers throughout his career. His mentorship was notable not only for the number of people he guided, but for the quality that he brought to each relationship. He engaged seriously with the work of his mentees, provided detailed and constructive feedback, and was genuinely invested in their development as scientists and in the trajectories of their careers. He was known to spend hours on a single conference presentation with a student, not because he was a perfectionist on their behalf in the way he was on his own, but because he wanted them to communicate their ideas as clearly and as well as possible. His group meetings were a kind of academic powwow: a freewheeling exchange of ideas that inspired all involved to pursue stronger work. He was also a close friend and confidante to many of his mentees. At scientific conferences, he was more likely to be found in animated conversation with his mentees at a nearby bar than attending official receptions.

Before it became a more widely shared value in the research community, Tom made deliberate efforts to mentor students from underrepresented groups, regularly recruiting summer interns from HBCUs and Latino-serving institutions. He took pride in the accomplishments of his mentees, many of whom went on to build independent research careers, in some cases supported by funding they first obtained by building on the tools and resources Tom’s group had developed and made freely available to the research community. His influence on the careers of those who trained with him, together with the broader community of researchers he inspired through his work and commitment to rigorous scientific inquiry, is perhaps the most durable part of his legacy. The collaborations that grew among these researchers continue to extend that legacy to this day.

As those who knew Tom would readily agree, he was also a singular personality. Although he was raised in a deeply Catholic Austrian-American milieu in small-town Minnesota, his life ultimately became remarkably cosmopolitan. He spent time in a Catholic seminary in Chicago as a teenager, served in Vietnam, studied Arabic in Egypt, and made Washington, DC, his home throughout his professional career. He was genuinely interested in people from different backgrounds, always eager to hear their stories and to share his own. He liked to say that he chose to study Arabic precisely because it was unconventional, a reflection of the independent streak that remained with him throughout his life. Even in his later years, he continued to jot down notes in Arabic script. He could move effortlessly from linguistics to religion, science fiction to Egyptology, or the architecture of his beloved DC, before arriving at the perennial disappointments of Washington’s sports teams. He was equally fond of a good beer and a lively debate, frequently combining the two at his favorite neighborhood Eritrean bar, where the conversation often continued until closing time and changing his mind was far less common than ordering another round. A child of the 1960s in outlook, he retained a healthy skepticism of authority throughout his life. While fiercely independent, he nevertheless believed deeply in the value of collaboration, recognizing that strong teams are built from individuals with distinct perspectives.

Tom Rindflesch leaves behind tools actively used by the biomedical research community, resources that continue to support discovery across clinical and translational science, and a generation of researchers shaped by his mentorship and his example. In the current era of large language models, the linguistically grounded, interpretable, incremental approach he championed can seem like a road not taken, a tradition swept aside by the sheer empirical force of data and scale. But the field is also increasingly recognizing the limitations of approaches driven by data and scale alone: the surge of neurosymbolic approaches, the growing demand for interpretability, and the stubborn difficulty of broad-coverage language understanding all suggest that the questions Tom spent his career asking are far from settled. Those of us who worked with him carry those questions forward, along with his high standards for serious, intellectually rigorous research. Those whose lives and work were touched by his example are invited to share tributes and reminiscences on the Kudoboard established in his memory (https://www.kudoboard.com/boards/hkCnDTie).

## Data Availability

No new data were generated or analyzed in support of this research.
